# Stereotactic Arrhythmia Radioablation Treatment of Ventricular Tachycardia: Current Technology and Evolving Indications

**DOI:** 10.3390/jcdd10040172

**Published:** 2023-04-17

**Authors:** Fabrizio Guarracini, Massimo Tritto, Antonio Di Monaco, Marco Valerio Mariani, Alessio Gasperetti, Paolo Compagnucci, Daniele Muser, Alberto Preda, Patrizio Mazzone, Sakis Themistoclakis, Corrado Carbucicchio

**Affiliations:** 1 Department of Cardiology, S. Chiara Hospital, 38122 Trento, Italy; 2 Electrophysiology and Cardiac Pacing Unit, Humanitas Mater Domini Hospital, 21053 Castellanza, Italy; m.tritto@libero.it; 3 Cardiology Department, General Regional Hospital F. Miulli, 70021 Acquaviva delle Fonti, Italy; a.dimonaco@gmail.com; 4 Department of Cardiovascular, Respiratory, Nephrology, Anaesthesiology and Geriatric Sciences, Sapienza University of Rome, 00100 Rome, Italy; marcoval.mariani@gmail.com; 5 Department of Cardiology, ASST-Fatebenefratelli Sacco, Luigi Sacco Hospital, University of Milan, 20157 Milan, Italy; alessio.gasperetti93@gmail.com; 6 Cardiology and Arrhythmology Clinic, University Hospital Ospedali Riuniti Umberto I-Lancisi-Salesi, 60126 Ancona, Italy; paolocompagnucci1@gmail.com; 7 Cardiothoracic Department, University Hospital, 33100 Udine, Italy; daniele.muser@gmail.com; 8 Department of Cardiac Electrophysiology and Arrhythmology, IRCCS San Raffaele Hospital, 20132 Milan, Italy; preda.alberto@hsr.it; 9 Cardiothoracovascular Department, Electrophysiology Unit, Niguarda Hospital, 20162 Milan, Italy; patrizio.mazzone@ospedaleniguarda.it; 10 Department of Cardiothoracic, Vascular Medicine and Intensive Care, Dell’Angelo Hospital, Mestre, 30174 Venice, Italy; sakis.themistoclakis@aulss3.veneto.it; 11 Department of Clinical Electrophysiology and Cardiac Pacing, Centro Cardiologico Monzino IRCCS, 20138 Milan, Italy; corrado.carbucicchio@ccfm.it

**Keywords:** ventricular tachycardia, electrical storm, stereotactic arrhythmia radioablation, STAR

## Abstract

Ventricular tachycardia in patients with structural heart disease is a significant cause of morbidity and mortality. According to current guidelines, cardioverter defibrillator implantation, antiarrhythmic drugs, and catheter ablation are established therapies in the management of ventricular arrhythmias but their efficacy is limited in some cases. Sustained ventricular tachycardia can be terminated by cardioverter-defibrillator therapies although shocks in particular have been demonstrated to increase mortality and worsen patients’ quality of life. Antiarrhythmic drugs have important side effects and relatively low efficacy, while catheter ablation, even if it is actually an established treatment, is an invasive procedure with intrinsic procedural risks and is frequently affected by patients’ hemodynamic instability. Stereotactic arrhythmia radioablation for ventricular arrhythmias was developed as bail-out therapy in patients unresponsive to traditional treatments. Radiotherapy has been mainly applied in the oncological field, but new current perspectives have developed in the field of ventricular arrhythmias. Stereotactic arrhythmia radioablation provides an alternative non-invasive and painless therapeutic strategy for the treatment of previously detected cardiac arrhythmic substrate by three-dimensional intracardiac mapping or different tools. Since preliminary experiences have been reported, several retrospective studies, registries, and case reports have been published in the literature. Although, for now, stereotactic arrhythmia radioablation is considered an alternative palliative treatment for patients with refractory ventricular tachycardia and no other therapeutic options, this research field is currently extremely promising.

## 1. Introduction

Endo-epicardial radiofrequency catheter ablation (RFCA) is an established treatment for patients with structural heart disease and recurrent ventricular tachycardia (VT) that is defined as ≥3 consecutive beats with a rate 100 b.p.m. originating from the ventricles, independent from atrial and atrioventricular nodal conduction [1,2]. Several randomized clinical trials demonstrated that RFCA significantly reduces VT recurrences, implantable cardioverter defibrillator (ICD) shocks, and electrical storm [2]. These positive effects also translate into a reduced rate of cardiovascular hospitalizations in patients submitted to RFCA [3]. However, this procedure undoubtedly has important limitations and drawbacks that significantly contribute to hampering its widespread application in clinical practice. In fact, the complexity and extent of the arrhythmogenic substrate decrease RFCA efficacy acutely and in the long term, especially in patients with non-ischemic cardiomyopathy [4]. Currently used radiofrequency (RF) energy settings might fail to steadily inactivate deep substrates. For several reasons, percutaneous epicardial access cannot be obtained in a significant proportion of patients, and, when it is achieved, RF energy delivery might be limited due to the risk of collateral injuries [5]. Despite relevant technological improvements, RFCA remains a long-lasting and rather complex procedure requiring extensive catheter manipulation within the ventricles and/or the pericardial space. As a result, the tolerability of the procedure might be limited, and the risk of a major complication is relatively high, especially in more compromised or frail patients. In particular, patients classified at high risk according to the PAINSED score have either a significantly higher probability of acute decompensation during RFCA [6] or long-term mortality [7].

Stereotactic arrhythmia radioablation (STAR) is a specialized procedure that uses advanced imaging techniques to accurately locate and target the areas of the heart arrhytmic substrate responsible for VT [8,9,10]. This technique involves the use of high-energy radiation beams to induce cellular apoptosis and fibrosis also in viable myocardium providing the electrophysiological substrate for VT occurrence. During the procedure, the patient is positioned on a specialized table and a series of detailed images are obtained using cardiac tomography or magnetic resonance scans. The images are then used to create a 3D map of the patient’s heart, which allows precise targeting of the arrhythmogenic area. Once the area has been identified, a specialized radiation therapy machine is used to deliver high-energy radiation beams to the targeted tissue. STAR is a minimally invasive procedure that is typically performed under conscious sedation. Patients typically return home the same day as the procedure, with a relatively short recovery period [11]. Hence, STAR represents an attractive alternative for patients with recurrent VT. In fact, since the lesion is achieved noninvasively and without the need for anatomical contact, this technique has the potential to overcome some RFCA limitations including the safety and the efficacy of ablation procedures in hazardous conditions or inaccessible substrates. While this procedure is still considered to be relatively new, initial studies have shown promising results in the treatment of VT, particularly in patients who have not responded to other forms of treatment, such as medication or traditional catheter ablation [12,13]. Long-term follow-up studies have also demonstrated promising outcomes, with sustained improvement in heart rhythm and quality of life [14,15]. However, further research is needed to fully evaluate the efficacy and safety of this technique, as well as to identify the optimal patient selection criteria and treatment protocols. Patient selection criteria for STAR typically involve a multidisciplinary evaluation by a team of cardiac electrophysiologists, radiation oncologists, and imaging specialists. The selection criteria can vary somewhat depending on the individual case and the medical center performing the procedure. General considerations are included in Table 1.

STAR has potential risks and limitations, and there are certain patient populations that may not be suitable candidates including those with conditions such as pregnancy, prior radiation therapy, active infection or inflammation, and unstable medical conditions [10,16]. STAR is an evolving field and ongoing research and technological advancements will possibly move its use also in the treatment of other arrhythmias such as atrial fibrillation. Moreover, advances in imaging techniques may improve the accuracy in targeting the areas of the heart responsible for arrhythmias, potentially leading to improved treatment outcomes and fewer complications as well as combination therapies with anti-arrhythmic drugs or catheter ablation. However, the current diffusion of STAR remains limited to a few specialized medical centers and hospitals, as it requires highly skilled and experienced medical professionals and specialized equipment. The small number of medical centers capable of treating these such complex patients is linked to the need to find both advanced skills in the ablative treatment of ventricular arrhythmias and advanced skills in terms of radiotherapy treatment of different pathologies. The close collaboration between these two clinical skills is the key to the success of radiotherapy treatment of ventricular arrhythmias unresponsive to standard treatments.

## 2. Physical Bases and Principles of Radiotherapy in Treatment of Ventricular Arrhythmias

Stereotactic radiotherapy is based on the physical phenomenon of ionization that happens when high-energy radiation, called ionizing radiation, breaks down chemical bonds removing electrons from atoms in the irradiated tissue, resulting in the generation of charged particles (ions) [17]. There are two types of ionizing radiation that can interact with matter: corpuscular radiation (light-charged particles such as electrons, positrons, and protons) and photons (X-ray and gamma-ray) [18]. On the one hand, the interaction of charged particles with matter is known as direct ionization, resulting from the direct collision of the particles with orbital electrons, which are forced to leave the parent atom. However, the interaction of high-speed electrons with matter may also result in the deflection of the original trajectory of the particle when it approaches the atomic nucleus, followed by the release of part of the electron’s kinetic energy as X-radiation, the so-called Bremsstrahlung phenomenon [19]. On the other hand, photons (X-rays and gamma-rays) do not have an electric charge and may transfer energy only in case of a direct hit with water molecules in order to generate reactive free radicals (via photoelectric effect, Compton effect, and pair production), which then interact with the target tissue in a process known as indirect ionization [18]. Though charged particles and gamma-rays are suitable energy sources, X-ray is the most used energy source for external-beam radiation therapy, and indirect ionization is the mechanism responsible for the radiation therapeutic effects. In clinical practice, the Bremsstrahlung process is used to generate X-ray beams for diagnostic or therapeutic purposes. In diagnostic radiology, low-energy X-ray beams are produced in a vacuum glass tube (called the X-ray tube) with an electric static field where a stream of electrons is accelerated from a cathode toward an anode (made of tungsten and molybdenum) resulting in electron energy conversion to photon beam. Unfortunately, low-energy X-rays are not suitable for radiotherapeutic applications because they do not penetrate deep into the tissue. For therapeutic purposes, dedicated linear accelerators (LINAC), are used to accelerate electrons to higher energy levels before hitting the target material, producing high-energy Bremsstrahlung X-ray beams that can penetrate the body depositing the energy in the targeted tissue [3]. Technological advances have led to the currently used stereotactic radiotherapy systems, such as the True Beam STX system (Varian Medical Systems, Palo Alto, CA, USA) and the CyberKnife (Accuray, Sunnyvale, CA, USA), in which the basic LINAC system has been implemented with image guidance, multi-leaf beam collimator, and robotic arm with rotational capabilities in order to reduce skin toxicity and off-target radiation, delivering multiple non-co-planar photon beams [20]. Compared to photon radiation, proton radiation shows potential advantages in terms of minimizing adjacent organ injury and improving target accuracy. The Bragg effect, also known as the Bragg peak, is a phenomenon observed in proton beam radiation where the energy deposited by protons increases as they slow down and approach the end of their path.This results in a higher dose delivered at a specific depth, known as the Bragg peak, compared to the surrounding tissues. This is in contrast to photon beam radiation, where the dose is delivered continuously throughout the tissue. Therefore, this effect allows for more precise targeting of tumors while minimizing damage to surrounding healthy tissue. This is because the depth at which the peak occurs can be adjusted by varying the energy of the proton beam [21]. The ionization process produced by high-energy beams changes the chemical and physical properties of cellular components, such as DNA or enzymes, and is responsible for irreversible damage eventually leading to cellular apoptosis. Indeed, the biological effects of ionization are mainly the results of double-strand DNA breaks [21,22], although oxygen free radicals cytotoxicity [23] and radiation-induced bystander effect (RIBE) may affect cell functioning and viability [24]. These biological effects of radiotherapy derive from studies focused on tumor biology, involving rapidly dividing cells. However, myocytes are not-dividing cells and the mechanism of injury in cardiac tissue is likely to be different than that for cancer and is not fully understood. The damage to cardiac tissue is probably multifactorial and characterized not only by DNA double-strand breaks and oxygen-reactive species cytotoxicity but also by microvascular injury and ischemic cell death [20]. In proof-of-principle animal studies, the radiotherapy effect started after several weeks or months and was associated with radiation-induced fibrosis in specimens collected 3–6 months after treatment [25,26,27,28]. The delayed treatment effect was confirmed by recent case reports [29,30] and supported by postmortem immunohistochemical analysis of morphologic changes in the myocardium after cardiac irradiation with a single dose of 25 Gy, showing myocardial cell death by apoptosis within the first months followed by fibrosis and elastosis [31]. However, observational data showed acute termination of the ventricular storm or rapid reduction of arrhythmia burden within days after therapy, suggesting the presence of an anti-arrhythmic mechanism of cardiac radiotherapy occurring earlier and different from conduction block due to radiation-induced fibrosis [10,32,33,34,35,36]. The radiobiological mechanisms mediating acute/subacute electrophysiological changes responsible for the anti-arrhythmic effect before the potential onset of fibrosis are incompletely understood. The ultrastructural changes found in human heart after 12 days from irradiation showed disruption of cellular machinery with damage and widening of intercalated discs and intercellular junctions, suggesting conduction block of action potential at cellular level as possible anti-arrhythmic mechanism [37,38]. Conversely, a recent study on irradiated murine hearts revealed radiation-induced reprogramming of cardiac conduction mediated by transient reactivation of Notch signaling pathway leading to increased Nav1.5 and connexin Cx43 expression and increased conduction velocity, likely functionally eliminating reentrant circuits [39,40,41,42,43,44]. Further studies are needed to unravel the electrophysiological mechanisms subtending the antiarrhythmic effects of cardiac radiotherapy [45].

## 3. Pre-Procedural Imaging and Workflow

### 3.1. Identification of Structural Aspects Related to VT

**Figure 1:**
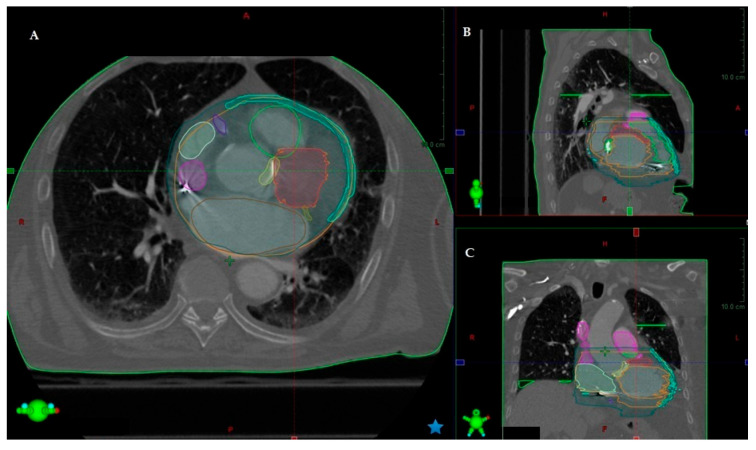
Treatment plan simulation. Contouring of the Planning Target Volume with respect to the intracardiac structures and the Organs at Risk is obtained through integrated “free-breathing” simulation-CT imaging. The Planning Target Volume in the axial (**A**), sagittal (**B**), and coronal (**C**) view, as delineated by the red contour line, is depicted.

One of the major challenges in STAR was developing a reliable target delineation method which is crucial for the treatment success. Electroanatomic mapping (EAM) in combination with delayed enhancement cardiac computed tomography (CT) was mainly used to characterize the arrhythmia substrate and to define the target area [46,47]. Cardiac CT performed with intravenous contrast (or oral contrast if deemed necessary to demonstrate gastrointestinal tract for inferior wall targets) is useful to define all the cardiac structures, in particular, regions of scar based on myocardial wall thickness and myocardial delayed enhancement. In general, myocardial wall thinning (<5 mm) and a delayed enhancement involving >50% of myocardial thickness identify transmural delayed enhancement (Figure 1).

Furthermore, high-density EAM performed in the endocardium and/or epicardium was used for an accurate 3D characterization of the left ventricle scar and all data obtained by EAM may be merged with the scar visualized on the CT scan [48,49,50]. Other data may be integrated to obtain an accurate target identification. A 12-lead ECG morphology of the VT helps to identify the exit of the electrical activity from the scar and can refine the area to target when the scar is defined on a CT scan [51]. Moreover, the cardiac magnetic resonance imaging technique of late gadolinium enhancement (LGE-CMR) enables the noninvasive detection of potentially arrhythmogenic substrates [52,53,54,55]. Beyond the detection of the presence of LGE, analysis of LGE characteristics enables the estimation of electrophysiological properties. In particular, regions of LGE with intermediate signal intensity represent areas of transition from normal myocardium to scar and may harbor critical components of re-entrant circuits for VTs. The strengths and limitations of the mentioned imaging modalities are reported in Table 2.

### 3.2. Mapping the VT

EAM remains the most accurate method of identifying the critical isthmus of the VT by integrating activation mapping during VT and pace-mapping during sinus rhythm.

Noninvasive electrocardiographic imaging during induced VT was reported to be able to obtain a precise map of the VT circuit [56,57,58]. For this procedure, patients wore a vest of 256 electrodes and underwent chest CT scanning. Electrocardiographic imaging maps were created to identify the site of earliest electrical activation during ventricle tachycardia (the “exit site”). A recent article reported that a workflow including computational ECG mapping is feasible, safe, and may improve stereotactic ablative radiotherapy [59].

All other tools can be used to plan a radiotherapy treatment [60]. A recent stereotactic arrhythmia radioablation was performed in a patient affected by an electrical storm in which catheter ablation was hampered due to left ventricular apical aneurism with thrombosis [61]. In order to determine the target myocardial tissue and treatment results, a merge study including VT morphology, cardiac-gated CT, myocardial 18F-Fluorodeoxyglucose positron emission tomography (18F-FDG PET-CT), and 99mTc-Sestamibi Gated single-photon emission computed tomography (SPET-CT) was performed to plan the final target area on the interventricular septum (Figure 1).

### 3.3. Further Methods to Improve Imaging Quality

**Figure 2:**
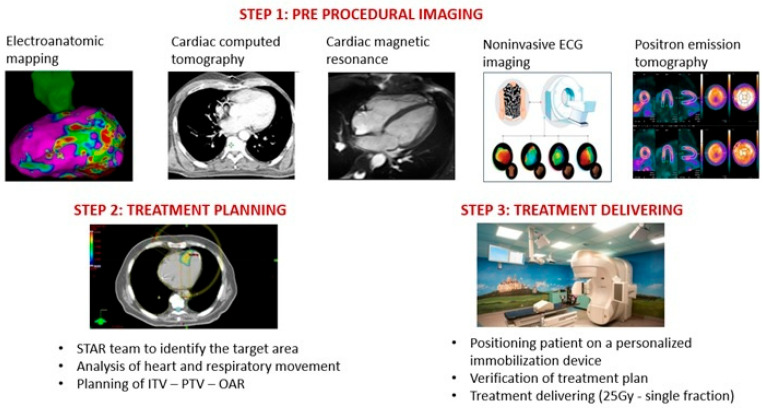
STEP 1, STEP 2, and STEP 3 interventional workflow for STAR. ITV (internal target volume), PTV (planning target volume), OAR (organs at risk).

Most patients with structural heart disease carry an ICD to prevent sudden cardiac death. The ICD lead motion may be useful to improve the treatment plan by providing further information on cardiac motion. A recent article reported the importance of evaluating the respiratory and cardiac-induced motion of an ICD lead during stereotactic arrhythmia radioablation of VT to improve treatment planning [62]. After collecting all the imaging data, the treatment plan is prepared with the collaboration of electrophysiologists, cardio-radiologist, radiation oncologists, and medical physicists [60]. For radiotherapy planning purposes, a 4-dimensional CT scan (usually 2 mm slice thickness) is usually obtained with patients in the supine position preferably using a personalized immobilization device. An internal target volume (ITV) is created to compensate for heart and respiratory movement (based on 4-dimensional CT) and the planning target volume (PTV) is built further expanding the ITV in 3 dimensions considering residual uncertainties caused by patient positioning and movements and by any displacement due to the heartbeat alone. Organs at risk (OAR) including lungs, healthy heart (heart minus PTV), left coronary, esophagus, spine, and ICD are delineated and excluded from treatment. Despite the presence of different treatment systems, usually, a dose of 25 Gy (single fraction) is prescribed to PTV with the patient in the immobilization cast [60]. The precise radiotherapy target delineation is crucial for the PTV trying to avoid unnecessary large treatment volumes as data suggest that relatively small irradiated volumes might be sufficient for VT reduction. However, the target delineation workflow is significantly different between studies and we cannot exclude that some of the treatment failure reported in the literature was due to suboptimal target delineation. Fortunately, some authors tried to challenge this problem by developing dedicated software for countering with an offline fusion of electroanatomic data with CT-reconstructed 3D models, which can later be imported to the radiotherapy planning system [63,64]. This software allows for higher precision than an indirect comparison between the EAM data and treatment planning CT. The Figure 2 reports a reasonable procedural work-flow to perform STAR.

## 4. Previous Experiences and Ongoing Studies

The use of STAR in humans was first described in a 2015 report by Loo et al., in which the technique successfully controlled ventricular tachycardia (VT) episodes in a 71-year-old male patient with ischemic cardiomyopathy, severely reduced ejection fraction, and oxygen-dependent chronic obstructive pulmonary disease [33]. Despite its relatively recent introduction, several studies have been conducted to date, enrolling approximately a total of a hundred patients, and providing guidance for the informed management of patients [11,65]. The main completed/ongoing studies on STAR are resumed in Table 3.

STAR established itself as a major breakthrough in the field of arrhythmia care in 2017 when the first series involving 5 very-high clinical risk patients (mean age, 66; New York Heart Association class III-IV; mean left ventricular ejection fraction, 23%) with refractory VT was published [56]. Patients were selected on a case-by-case basis, after having experienced at least three VT episodes in the preceding three months despite having received two or more antiarrhythmic drugs and having undergone an invasive catheter ablation procedure, or having contraindications to catheter ablation (n = 2). The authors combined noninvasive VT mapping with electrocardiographic imaging, and myocardial substrate characterization with a myocardial radionuclide (n = 4) or cardiac magnetic resonance (n = 1) imaging, to delineate the clinical target volume. STAR was performed with photon radiation (TrueBeam, Varian Medical Systems, Palo Alto, CA; 25 Gy over 14 min; median target volume, 49 mL), and led to a very significant (99.9%) reduction in VT burden after the initial six-week blanking period.

These highly promising results prompted the execution of the ENCORE-VT trial by the same group; the study results were published in 2019 [36]. In the ENCORE-VT trial, a total of 19 patients (i.e., the largest patient sample to date) with either three or more sustained monomorphic VT episodes in the last three months (n = 17) or premature ventricular contractions (PVC)-related cardiomyopathy with monomorphic PVC burden >20% (n = 2) despite having received one or more antiarrhythmic drugs and having undergone one or more invasive catheter ablation procedure (or having contraindications for the invasive procedure, n = 3) were enrolled. The preprocedural workflow was similar to that used in the 2017 paper (ECG imaging, myocardial radionuclide imaging, cardiac magnetic resonance), and STAR was performed with a single dose of 25 Gy over a median of 15 min, targeting the arrhythmogenic region neighboring the VT exit site (median target volume, 25 mL). The authors reported a 94% reduction in VT burden compared to baseline after the initial six-week blanking period and a significant reduction in the PVC burden among the two patients with PVC-related cardiomyopathy. Of note, recurrent VT was observed in 69% of patients after the blanking period. These results were accompanied by an improvement in several quality-of-life measures, and a reduction in the use of antiarrhythmic medications (especially combined antiarrhythmic drugs use). Several radiation-related adverse events were reported, including one pericarditis, two grade 2 radiation-induced pneumonitis, and 5 asymptomatic pericardial effusions. Notably, three patients died for recurrent VT after a mean of 10 months following STAR, raising concerns about its durable efficacy, and/or the value of targeting the VT exit site (in STAR) as opposed to the VT isthmus (in invasive catheter ablation).

Still, in 2019, Neuwirth et al. reported on the safety and efficacy of STAR in 10 patients after failed invasive catheter ablation. A different method was used for the delineation of the target volume, i.e., regions of interest were identified during the prior invasive electrophysiological procedure by means of voltage (with the CARTO electroanatomical mapping system, Biosense Webster, Irvine, CA, USA), pace, and entrainment mapping [66]. Contrast-enhanced computed tomography was used to delineate the clinical target volume, using the implantable defibrillator lead as a surrogate marker for compensation of respiratory movements. STAR was delivered with the CyberKnife (Accuray Inc., Sunnyvale, CA, USA), which is a robotic system allowing more precise and respiration-gated radiotherapy administration, resulting in a very limited 22 mL mean target volume; STAR was delivered in a single session using 25 Gy over a mean of 68 min. Although VT recurred in 8/10 patients after the initial 3 month blanking period, an 86% reduction in VT burden was reported. Three patients died of non-cardiovascular causes. These results should be interpreted after considering that amiodarone administration was interrupted right before STAR in this study, as opposed to previously cited reports. The only acute adverse event was nausea, while the sole long-term potentially concerning adverse event was a progression in the severity of mitral regurgitation in one patient, which was noted 17 months after treatment.

In 2020, Gianni et al. reported on the long-term (mean follow-up, 12 ± 2 months) efficacy of STAR [67]. They enrolled 5 patients with structural heart disease and recurrent VT despite one or more prior invasive catheter ablations and one or more prior antiarrhythmic drugs. The clinical target volume (mean value, 143 mL) was selected as the region showing reduced thickness at contrast-enhanced cardiac CT, after correlating CT images with both low-voltage regions at prior invasive electroanatomical voltage mapping (CARTO, Biosense Webster, Irvine, CA, USA), and 12-lead ECG VT morphology, resulting in a less extensive preprocedural assessment compared to prior experiences. STAR (mean, 2.7 Gy over a mean of 82 min) was again delivered with the Cyberknife robotic system, using a transvenous active-fixation trans-jugular pacemaker lead as a respiration gating reference. Although a marked reduction in VT burden was observed in 4/5 patients in the first 6 months, by the end of follow-up, VT burden increased, requiring reinstatement of antiarrhythmic drug treatment in all cases, and repeat invasive catheter ablation in 3 patients. The long-term failure was attributed to several factors, including inadequate radiation dosing, technical issues resulting in adequate radiation coverage of the target volume, and the high-risk clinical profile of enrolled patients.

More recently, the preliminary results in 8 patients of the Italian STereotactic RadioAblation by Multimodal Imaging for Ventricular Tachycardia (STRA-MI-VT) study have been published [12,46]. Patients were enrolled for refractory VT (at least 3 VT episodes; n = 5 patients with electrical storm) despite any pharmacological/non-pharmacological treatment; in all patients invasive catheter ablation or any invasive procedure was contraindicated (i.e., due to intracardiac thrombus mitral-aortic mechanical prosthesis, etc.) and radiotherapy was considered as a bailout treatment. The target volume for STAR was mainly chosen based on the location of fibrosis, as identified by delayed-enhancement cardiac CT (in all patients), and electroanatomical voltage mapping with the CARTO system (available in 5 patients). A single 25-Gy radioablation dose was delivered by a linear accelerator-based volumetric modulated arc therapy technique to a mean clinical target volume of 39 mL over 31 min; the reduction in recurrent VT burden was significant at 3 months and close to significance at 6 months, with the limitation of the sample size. The authors reported also a significant reduction in ICD shocks, as well as the abolition of electrical storms. No treatment-related serious adverse events were observed over the median follow-up of 8 months, the three reported deaths being attributed to non-cardiovascular causes. The completion of the STRA-MI-VT study, with a planned enrollment of 15 patients and a complete 12-month follow-up, will further inform several still uncertain aspects of STAR, including the complex patient selection process and the optimal pre-procedural imaging protocol, with special emphasis on the possibility to exclude cardiac magnetic resonance in such a special cohort of complex and clinically unstable ICD carriers.

## 5. Short- and Long-Term Safety Data

One of the main concerns associated with the use of radiotherapy for the management of refractory VTs is its safety. Potential adverse events (AEs) of this treatment can in fact present as cardiac AEs, due to the direct myocyte damage caused, or as extra-cardiac AEs, from the irradiation of the surrounding anatomical structures (lungs, esophagus, and mediastinum).

Unfortunately, AEs associated with radiotherapy are not uncommon. They have been observed in most studies addressing the topic [10,32,33,34,35,36,66,73,74,75], with the highest rate of AEs being reported in the ENCORE-VT trial, where 88% of patients reported treatment-associated AEs [36]. The severity of the majority of AEs in the ENCORE-VT trial, however, as well as across different studies from multiple groups, has been graded as “mild” or “moderate”. Reassuringly, when only severe AEs requiring hospitalization/active management are considered, the overall rates are significantly lower (22% in the ENCORE-VT experience).

In the assessment of radiotherapy safety, an important time dependency of AEs has been described. Different AEs may arise at different time points during follow-up. A common way of reporting AEs has been classifying them as acute AEs (within 48 h of intervention), short-term AEs (2 to 90 days), or long-term follow-up AEs (90 days to 12 months). In an acute setting, the most commonly reported non-cardiovascular side effects are nausea and vomiting, followed by fatigue and, less commonly, hypotension [12,36,66]. Crucially, no acute adverse effects to the indwelling leads of ICDs have to date been reported. This safety finding is of paramount importance, given the extremely high rate of patients referred for radiotherapy with an ICD in place. In multiple studies, however, an increased arrhythmic activity leading to VT storms immediately after the delivery of radiotherapy has been observed, with an overall incidence rate of around 7% [34,73,75]. Furthermore, as reported by a recent systemic review on the topic [65], these post-radiotherapy VT storms events may even have been underreported, due to the presence of a blanking period of 6- to 12- weeks in the outcome assessing protocol of most other studies [10,33,36,66]. The actual incident rate may therefore be higher. Considering the often highly compromised status of patients undergoing radiotherapy, these events may take a heavy clinical toll on patients and it is necessary for the managing team to be prepared to quickly react to a similar event. A mixture of AEs has been described at short- and long-term follow-up assessments. While left ventricular ejection fraction (LVEF) was consistently shown as stable across studies reporting multiple pre- and post-radiotherapy LVEF assessments [10,12,32,33,36,65,66,76], heart failure exacerbations and pulmonary edema were not infrequent within the first 90 days of follow up [36,76,77]. In prospective assessments, pericarditis, pneumonitis, pneumonia, stroke, and pulmonary embolism episodes were also reported, with a combined incidence rate of 23.5% (4/17 patients) in the ENCORE VT and of 12.5% (1/8) in the STRAMI-VT study, respectively [12,36]. One patient on the ENCORE-VT trial developed a grade 3 gastropericardial fistula 2-year after treatment. The observed 1-year mortality post-radiotherapy is high, ranging from 5% to almost 50%, depending on the study cohort. Many of the causes of death observed, however, are either of non-cardiac origin or not related to radiotherapy. Patients often undergo radiotherapy for VT management as a palliative/bailout option when their underlyng cardiac condition is already very advanced. In a competitive risk analysis, the attributable risk of mortality from radiotherapy compared to their baseline condition would probably be very low.

It is however important to remember that while the risk profile of this technique is acceptable when it is used as a palliate therapy, a different risk-benefit assessment is required for patients who may have a longer survival expectancy. Radiotherapy is in fact well known to also have delayed effects (often after years) on cardiac structures, potentially affecting coronary arteries, the conduction system, and valvular structures [78]. Additionally, albeit radiotherapy being delivered with systems designed to minimize the toxicity to surrounding structures, chest, and cardiac motion also increase the risk of an off-target delivery. Potential non-cardiac delayed side effects of this treatment, therefore, include pulmonary fibrosis, esophageal ulceration, tracheobronchial fistula, and neoplasms. These AEs generally become visible over long periods of time and when multiple irradiations are delivered. Results from animal models have reported the occurrence of similar complications [79,80]. In humans, currently reported delayed AEs are limited to a gradual progression of mitral valve regurgitation 17 months after radiotherapy delivery in the ENCORE-VT trial [36]. However, follow-up assessments beyond 12 months are scarce [10,66], because of the novelty of this treatment and the relatively high mortality rate that patients considered clinically eligible for radiotherapy face due to the severity of their condition. Safety data for the use of radiotherapy for VT management are therefore temporally still limited. As per the recent consensus statement, further studies are required to properly assess the long-term impact of this treatment, and before an expansion of clinical indications behind this treatment modality can be implemented [8]. Continuous optimization of the technology and the strategies behind radiotherapy is also of paramount importance to improve the safety of this technique. In several preclinical studies was demonstrated that a dose of at least 25 Gy was sufficient to create fibrosis in the heart similar to catheter ablation [80,81].

Clearly determining the minimal radiation dose level effective in VT suppression may help reduce the current average prescribed dose (≈25 Gy), which is relatively high compared to the one used for the management of other diseases (i.e., 20 Gy for arterio-venous malformation [82] or 18 Gy for seizure [83]).

Reducing radiation dose to the minimal effective dose, the amount of surrounding tissue affected will be reduced as well, lowering the probability of long-term complications [84]. Table 4 summarizes the possible organ complications related to STAR [68,69,70,71,72,85].

## 6. Conclusions

Based on current published experience on success rates and complications, STAR should not be used in place of conventional catheter ablation. However, STAR represents an exciting advancement in the treatment of recurrent VT, offering patients irresponsive to conventional therapies a minimally invasive and effective treatment option even if the routinary use is limited to hospitals equipped with appropriate technology and expertise. Further research is needed to fully evaluate the efficacy and safety of this technique, as well as to identify the optimal patient selection criteria and treatment protocols.

## Figures and Tables

**Table 1:** General patient selection criteria for STAR. VT (ventricular tachycardia), CT (cardiac tomography), MRI (magnetic resonance), 3D (three-dimensional).

Indication	Comment
Challenging location of the tachycardia	Patients with VT that originates from a specific, localized area of the heart, which is not possible or very challenging to achieve with catheter ablation.
Contraindications to catheter ablation	Inaccessible anatomical sites, inability to access the heart itself, or inability for a patient to tolerate a catheter ablation procedure
General health and medical history	Patients who are in generally poor health, with significant comorbidities, which would be considered at high risk for a transcatheter approach.

**Table 2:** Strengths and limitations of imaging modalities to identify structural aspects related to VT.

Method	Strengths	Limitations
Cardiac CT	Identification of coronary artery disease assessing left ventricular anatomy and scar burden	Radiation exposure Risk of contrast-induced nephropathy and allergic reaction Low accuracy in detecting small fibrous areas of scar tissue
Cardiac MRI	Highly spatial resolution and detailed 3D images of the heart Comprehensive assessment of myocardial tissue by use of multiparametric approach (T1 and T2 mapping, late gadolinium enhancement, and diffusion-weighted imaging) Quantitative assessment of myocardial scar	Significant time commitment required Contraindicated in patients with certain types of implanted devices Interpretation challenges
Nuclear imaging	Detection of areas of hypoperfusion or scar that may not visible on other imaging modalities Study of myocardial viability	Expensive Low spatial resolution and sensitivity Radiation exposure
Electroanatomical mapping	Real-time mapping of cardiac electrical activity in both endocardium and epicardium Detailed 3D images of the heart Accurately identification of VT circuit	Invasive catheterization Technical expertise

**Table 3:** Summary of main studies on STAR. LVEF (left ventricular ejection fraction), NYHA (New York Heart Association), VT (ventricular tachycardia), STAR (Stereotactic arrhythmia radioablation).

Reference	Cuculich et al. [10]	Robinson et al. [36]	Neuwirth et al. [66]	Gianni et al. [67]	Lloyd et al. [35]	Yugo et al. [68]	Chin et al. [69]	Carbucicchio et al. [12]	Lee et al. [70]	Qian et al. [71]	Kurzelowski R et al. [72]
Publication year	2017	2019	2019	2020	2020	2021	2021	2021	2021	2022	2022
Study design	Case series	Prospective	Case series	Prospective	Retrospective	Case series	Retrospective	Prospective	Prospective	Prospective	Case series
	Single center	Single center	Single center	Dual center	Single center	Single center	Single center	Single center	Three centers	Single center	Single center
No. of enrolled patients	5	19	10	5	10	3	8	7	7	6	2
Male—n (%)	4 (80)	17 (89.5)	9 (90)	5 (100)	7 (70)	2 (69)	8 (100)	7 (100)	4 (57)	6 (100)	2 (100)
Age range	66 (60–83)	66 (49–81)	66 (61–78)	62	61 (51–78)	72 (65–83)	75 ± 7.3	70 ± 7	60s–70s	72 (70–73)	69–72
Ischemic heart disease—n (%)	2 (40)	11 (57.9)	8 (80)	4 (80)	4 (40)	0	4 (50)	3 (43)	5 (71.4)	6 (100)	2 (100)
Non-ischemic Cardiomyopathy—n (%)	3 (60)	8 (42.1)	2 (20)	1 (20)	6 (60)	3 (100)	4 (50)	4 (57)	2 (28.6)	0	0
LVEF (%)	23 (15–37)	25 (15–58)	26.5 ± 3.2	34	/	20–59	21 ± 7	27 ± 11	27	20 (16–25)	20–22
NYHA class- %											
I		5.3		20		69		29		/	0
II		21.1	60	80	/	33		71	43	/	0
III	20	52.6	40				62.5		43	/	100
IV	80	21.1					37.5		14	/	0
Radiation type	Linear accelerator	Linear accelerator	Cyberknife	Cyberknife	Linear accelerator	Linear accelerator	Linear accelerator	Linear accelerator	Linear accelerator	Linear accelerator	Linear accelerator
Dose (Gy)	25	25	25	25	25	25	22.2 ± 3.6	25	25	25	25
Treatment time (min)	14	15 (5–32)	68 (45–80)	82 (66–92)	/	/	18 ± 6	31 ± 6	38	14 (11–15)	13
Average follow up	12 months	6 months	28 (16–54) months	12 ± 2 months	174 (118–273) days	2–54 weeks	234 (145–299) days	4 pt complete 6 months FU	6 months	231 (212–311) days	6 months
VT burden reduction	99.9%	94%	87.6%	No reduction	69%	61%	80%	93%	85%	31%	Sustained VT abolition after blanking
Complications related to STAR	1 stroke (not crearly related)	1 pericarditis; 1 heart failure (possible)	1 nausea 1 progression of mitral regurgitation	none	2 pneumonitis	none	none	1 nausea/vomiting 1 pulmonary fibrosis	none	1 pneumonitis 1 heart failure 1 moderate pericardial effusion	1 heart failure exacerbation And concomitant pulmonary embolism

**Table 4:** Possible STAR-related organ complications.

Irradiated Organ	Possible Complication	Symptoms
Heart	Arrhythmias, pericarditis, myocarditis, myocardial and valvular fibrosis, coronary atherosclerosis	Chest pain, palpitations, symptoms of heart failure
Lung	Pneumonitis and fibrosis leading	Cough, dyspnea, chest pain
Esophagus	Esophagitis and perforation	Dysphagia, chest pain, cough
Spinal cord	Inflammation and fibrosis	Sensory and motor dysfunction, paralysis

## Data Availability

Not applicable.
